# Postprandial triglycerides and fibroblast growth factor 19 as potential screening tools for paediatric non-alcoholic fatty liver disease

**DOI:** 10.1111/ijpo.13007

**Published:** 2023-02-03

**Authors:** Christina M. Sciarrillo, Kevin R. Short, Bryant H. Keirns, Destinee C. Elliott, Stephen L. Clarke, Sirish Palle, Sam R. Emerson

**Affiliations:** 1 Department of Nutritional Sciences, Oklahoma State University, Stillwater, Oklahoma, USA; 2 Department of Pediatrics, University of Oklahoma Health Sciences Center, Oklahoma City, Oklahoma, USA

**Keywords:** non-alcoholic fatty liver disease, paediatric obesity, postprandial triglycerides, steatosis

## Abstract

**Background:**

Better screening tools for paediatric NAFLD are needed. We tested the hypothesis that the postprandial triglyceride (TG) and fibroblast growth factor 19 (FGF19) response to an abbreviated fat tolerance test (AFTT) could differentiate adolescents with NAFLD from peers with obesity and normal weight.

**Methods:**

Fifteen controls with normal weight (NW), 13 controls with obesity (OB) and 9 patients with NAFLD completed an AFTT. Following an overnight fast, participants consumed a high-fat meal. TG and FGF19 were measured at baseline and 4 h post-meal. Liver steatosis and fibrosis were measured via Fibroscan.

**Results:**

Fasting TG and FGF19 did not differ among groups; 4 h TG in the NAFLD and OB groups were greater (197 ± 69 mg/dL; 157 ± 72 mg/dL, respectively) than NW (105 ± 45 mg/dL; *p* < 0.05) and did not differ from one another. Within the entire cohort, 4 h TG were stratified by high and low steatosis. Adolescents with high steatosis had 98% greater 4 h TG than adolescents with low steatosis. 4 h FGF19, but not fasting FGF19, was higher in children with low steatosis compared with high steatosis (*p* < 0.05). Using area under the receiver operating curve (AUROC), the only biochemical outcome with diagnostic accuracy for NAFLD was 4 h TG (0.77 [95% CI: 0.60–0.94; *p* = 0.02]).

**Conclusions:**

The postprandial TG response is increased in adolescents with obesity with hepatic steatosis, with or without NAFLD. Our preliminary analysis demonstrates 4 h TG differentiate patients with NAFLD from those without, supporting a role for the AFTT as a screening tool for paediatric NAFLD.

## 1 ∣ INTRODUCTION

Non-alcoholic fatty liver disease (NAFLD) is characterized by liver fat accumulation, accompanied by different degrees of fibrosis, inflammation and necrosis. As such, NAFLD is an array of liver diseases ranging from simple steatosis to non-alcoholic steatohepatitis (NASH). NAFLD has been recognized as the most prevalent chronic liver disease worldwide and can progress to cirrhosis and increase risk for hepatocellular carcinoma (HCC).^1^ A recent analysis of National Health and Nutrition Examination Survey (NHANES) data from 2017 to 2018 revealed that NAFLD (measured via transient elastography using Fibroscan) was present in 40% of adolescents and young adults.^2^ One of the hallmarks of the development of NAFLD is insulin resistance. Not surprisingly, NAFLD has been described as the liver's display of metabolic syndrome, as conditions including type 2 diabetes, dyslipidaemia and obesity commonly accompany a NAFLD diagnosis.^3^

Despite recent advancements in understanding the pathophysiology and clinical representation of NAFLD, screening tools for early stages of triglycerides accumulation are lacking. Currently, screening for NAFLD often relies on liver biochemistries (e.g., alanine amino transferase [ALT]), which are non-specific to NAFLD, so much so that the American Gastroenterological Association advises against their use for screening for NAFLD.^4^ Liver biopsy is the gold standard for the diagnosis of NAFLD, but it is expensive, invasive and not a feasible screening tool. Alternatives to liver biopsy include ultrasonography and MRI, but these methods are expensive and not widely available.

Screening for NAFLD in children and adolescents is critical. Children often have a unique fibrosis pattern relative to adults, characterized by portal fibrosis and inflammation. Notably, at the point of diagnosis, 15% of children have already advanced to stage 3 or 4 fibrosis.^5^ Thus, paediatric NAFLD is characterized by a distinctive pathophysiology that may be more aggressive and severe when compared with adults.^4,6^ Since adolescents often carry this burden of risk into adulthood, early detection tools for NAFLD in children are needed. One potential avenue for the early detection of liver steatosis is the assessment of postprandial triglycerides. Specifically, an adverse postprandial triglyceride response has been observed in children with NAFLD and may serve as one etiological mechanism leading to triglyceride accumulation in the liver.^7^ In fact, children with NAFLD exhibit a steep increase in triglycerides in response to a high-fat meal (HFM) and this rise is persistent throughout the postprandial period, lasting longer than children with obesity and normal weight without NAFLD.^7,8^ Moreover, postprandial hypertriglyceridemia in the context of NAFLD is closely related to insulin resistance and its subsequent metabolic dysfunctions, and hypertriglyceridemia is associated with the degree of hepatic steatosis, fibrosis and inflammation.^9-11^ However, obesity alone can promote postprandial hypertriglyceridemia.^12^ Because more than half of people with NAFLD have obesity, it is important to determine the ability of postprandial triglycerides to discriminate between children with obesity without NAFLD and children with NAFLD. However, previous work investigating postprandial hypertriglyceridemia in paediatric NAFLD utilizes a resource and time-intensive assessment protocol, characterized by repeated blood sampling and lasting up to 6–8h and a test meal containing quantities of total fat that may not mount a considerable postprandial triglyceride response.^13^ The abbreviated fat tolerance test, consisting of a fasting blood sample and a 4-h postprandial blood sample, is a more feasible approach, but has yet to be utilized to assess postprandial hypertriglyceridemia in paediatric NAFLD.

Another promising biomarker, fibroblast growth factor 19 (FGF19), is an endocrine signal linked to postprandial dyslipidemia in the context of NAFLD, where it has emerged as a novel intestinally derived regulator of hepatic triglyceride metabolism.^14^ Specifically, FGF19 regulates key features of triglyceride metabolism that contribute to the progression of NAFLD, including promoting fatty acid oxidation, suppressing de novo lipogenesis (DNL), increasing the export of triglycerides from the liver, and reducing inflammation, cytotoxicity and endoplasmic reticulum (ER) stress.^15^ FGF19 production and signalling are altered in people with NAFLD and closely related to hepatic triglyceride metabolism.^16,17^ Recent work demonstrated that children with obesity and NAFLD have lower fasting FGF19 compared with children with obesity without NAFLD.^17^ However, postprandial FGF19 has not been studied in children with obesity with and without NAFLD. Since FGF19 is a postprandial hormone, relying only on fasting FGF19 concentration to screening for paediatric NAFLD may underestimate the number of cases. Therefore, the objectives of this study were to evaluate the sensitivity and specificity of postprandial serum concentrations of triglycerides and FGF19, following an abbreviated fat tolerance test (AFTT), to predict the presence of NAFLD in adolescents.

## 2 ∣ METHODS

### 2.1 ∣ Participants

In a case–control pilot study, we enrolled three groups of participants (13.0–20.9 years old): NAFLD cases with obesity (*n* = 9 [NAFLD]), controls with obesity without diagnosed NAFLD (*n* = 13 [OB]) and controls with normal weight without diagnosed NAFLD (*n* = 15 [NW]). NAFLD cases were recruited from patients in the Section of Gastroenterology, Department of Paediatrics. NAFLD cases were adolescents with confirmed presence of NAFLD via liver biopsy, elevated liver enzymes and BMI ≥30 kg/m^2^ or ≥95th percentile (depending on their age). A diagnosis of NAFLD was made after the exclusion of other competing liver diseases, including autoimmune or metabolic liver diseases, Wilson disease and viral hepatitis. Further, patients with diabetes mellitus, genetic syndromes, kidney disease, hematologic disease and cardiovascular disease were excluded. NAFLD was ruled out in OB and NW via Fibroscan owing to the unethical nature of subjecting non-referred children to liver biopsy. Therefore, both OB and NW groups had not been diagnosed with NAFLD prior to study enrolment. For all participants <20 years old, BMI was based on growth curves and percentiles.^18^ For all participants 20.0–20.9 years old, BMI was based on the adult BMI formula (kg/m^2^). Normal weight was defined by BMI of 18.5–24.5 kg/m^2^ or within 5th to 85th percentile and obese was defined by BMI of ≥30 kg/m^2^ or ≥95th percentile. Participants in all groups were excluded if they were engaged in sports or other structured exercise on more than 3 days/week for the past 3 months. A thorough medical and social history was conducted with all participants prior to enrolment to rule out excessive use of alcohol or tobacco. Written informed assent and consent were obtained from participants and from parent/guardians of participants <18 years prior to study initiation, in accordance with the Institutional Review Board, which approved the study.

### 2.2 ∣ Overview of protocol

Participants completed one visit to the laboratory for testing. On the day prior to the study visit, participants were instructed to avoid vigorous physical activity, begin fasting 10 h prior to their study visit and sleep ≥7 h. Participants' weight, height and waist and hip circumferences were measured via calibrated stadiometer, balance and tape measure, respectively. Body composition was measured using dual energy X-ray absorptiometry (DXA) (GE/Lunar iDXA, GE-Healthcare, Fairfield, CT) to determine total body and regional fat and lean tissue. Liver steatosis and fibrosis were determined in all study participants with the Fibroscan 502 Touch (Echosens; Paris, France). The AFFT was then completed as described below.

#### 2.2.1 ∣ AFTT

The AFTT is a metabolic test that is a simplified version of a standard oral fat tolerance test and is used to quantify postprandial triglycerides. We previously determined that the AFTT is valid and reliable for measuring postprandial triglycerides.^19,20^ Before the meal, an IV catheter was placed by a registered nurse and a baseline blood draw was collected. Participants then consumed a high-fat meal and 4-h later a second blood draw was collected to quantify the postprandial triglyceride and FGF19 response. The high-fat meal consisted of coconut cream, chocolate syrup and vegan protein powder and was scaled to body weight (9 kcal/kg; 70% fat [92% saturated fat], 21% carbohydrate, 10% protein). This meal has been used in previous studies from our laboratory to assess its reliability and test the metabolic health of adults.^20-22^

### 2.3 ∣ Measurements

Fibroscan uses a transient elastography probe placed over an intercostal space to non-invasively measure liver stiffness as an estimate of fibrosis. For children and adolescents, the stiffness threshold for fibrosis stage F0 (none) versus F1 in children is 6.5 kPa.^23^ Liver steatosis is estimated from the controlled attenuation parameter (CAP). A CAP value of 225 dB/m defines the threshold between stage 0 steatosis (<5% fat by volume) and stage 1 or higher.^24^ Brachial blood pressure was measured using a standard blood pressure cuff on the upper arm using the SphygmoCor XCEL instrument (AtCor Medical, Inc., Itasca, IL).

### 2.4 ∣ Laboratory values

Whole blood collected at baseline was used to measure triglycerides, total-cholesterol (C), VLDL-C, LDL-C, HDL-C and liver enzymes (ALT, aspartate aminotransferase [AST]) using a Piccolo Xpress clinical chemistry analyser (Piccolo, Abbott, Princeton, NJ) and haemoglobin A1c (HbA1c) (Siemens DCA Vantage Analyser, Malvern, PA). The triglyceride measurement was repeated at 4 h after the meal. Serum aliquots collected at baseline and 4 h were stored at −80°C for later analysis. We measured fasting and 4 h FGF19 (Invitrogen, Human FGF19 ELISA), fasting insulin (Alpco, Insulin ELISA), fasting glucose (FujiFilm-Wako) and fasting adiponectin (Invitrogen, Human Adiponectin ELISA). The updated homeostasis model assessment (HOMA2) was used to estimate beta cell function (%B), insulin sensitivity (%S) and insulin resistance (IR) from fasting serum insulin and glucose concentrations.^25^

### 2.5 ∣ Statistical analyses

Data were checked for normality prior to analyses (skewness ≤2, kurtosis ≤7). When data were not normally distributed, corresponding non-parametric analyses were used or the ROUT method was used to identify and remove outliers. A general linear model was used to compare differences in participant characteristics across groups. A general linear model with repeated measures was used to compare the postprandial response for triglycerides and FGF19 to an AFTT between NAFLD, OB and NW. When significant interactions or main effects were present, post hoc analyses using Tukey HSD test were used to determine pairwise differences.

A general linear model was used to compare the total area under the curve (tAUC) and change from baseline to 4 h postprandial (Δ) for triglycerides and FGF19 across groups; post hoc analyses using Tukey HSD test were used to determine group differences. An unpaired *t*-test was used to determine differences in fasting and postprandial triglycerides and FGF19 and fasting insulin between participants with and without steatosis and/or fibrosis, respectively. Pearson correlations or Spearman correlations were used to determine the relationships between independent and dependent variables. The diagnostic accuracy of fasting and postprandial triglycerides and FGF19 was determined by evaluating the area under the receiver operating characteristic (ROC) curve (equivalent to c-statistic). The dichotomous categorical variable classified participants into two groups: participants with diagnosed NAFLD and participants free of NAFLD. Cohen D was calculated for postprandial triglycerides and FGF19. A *p*-value of <0.05 was considered statistically significant for all statistical tests. Analyses were conducted using GraphPad Prism (GraphPad Prism Inc.; La Jolla, CA).

## 3 ∣ RESULTS

### 3.1 ∣ Participant anthropometrics, demographics and characteristics

Participant characteristics are displayed in Table 1. NAFLD and OB exhibited significantly greater body mass, BMI, body fat %, android body fat, gynoid body fat and trunk fat compared with NW (*p* < 0.05), although NAFLD and OB did not differ for these measures. Systolic blood pressure was greater in obese than NW (*p* = 0.01); there were no other group differences in blood pressure (*p* > 0.05). NAFLD participants displayed a significantly higher liver fibrosis score than both OB (mean difference 4.8 kPa, *p* = 0.0008) and NW (mean difference 5.1 kPa, *p* = 0.0003). The fibrosis scores for OB and NW did not differ (*p* = 0.94). NAFLD patients had a significantly higher CAP score (i.e., steatosis) than NW (mean difference = 137.7 dB/m, *p* < 0.0001), but NAFLD did not differ from OB (mean difference = 43.5 dB/m, *p* > 0.05). There was a significant difference in CAP score between OB and NW (mean difference = 94.2 dB/m; *p* < 0.0001).

### 3.2 ∣ Metabolic and liver biochemistries

Fasting values for blood biochemistry are displayed in Table 2. There were no group differences in total cholesterol or LDL-C (*p* > 0.05). NAFLD participants exhibited lower HDL-C than normal weight controls (mean difference = −19 mg/dL; *p* = 0.003), as did OB (mean difference = −18 mg/dL; *p* = 0.001). NAFLD participants had significantly higher VLDL-C than NW (mean difference = 10 mg/dL; *p* = 0.03). The OB had a higher HbA1c compared with NW (*p* = 0.03), however NAFLD did not differ from OB or NW (*p* > 0.05). NAFLD and OB displayed significantly higher fasting insulin compared with NW (*p* < 0.0001), however NAFLD and OB did not differ (*p* > 0.05). Across groups, there was no difference in adiponectin concentration (*p* = 0.19). ALT and AST were higher in the NAFLD group than both control groups (*p*'s < 0.05) but did not differ between OB and NW (*p*'s > 0.05).

### 3.3 ∣ Fasting and postprandial triglycerides

**FIGURE 1:**
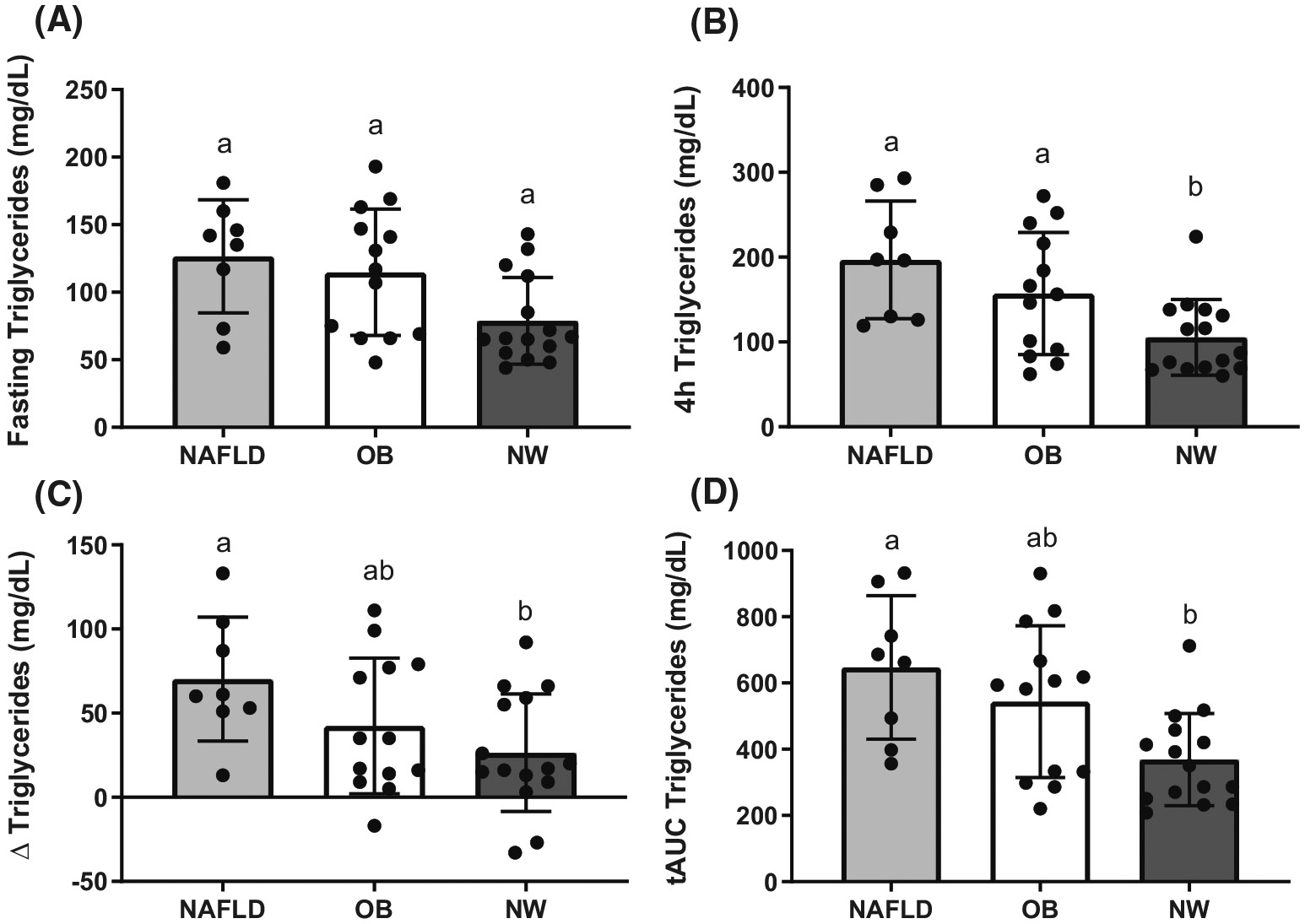
(A–D) Triglyceride parameters. (A) Fasting triglycerides during the AFTT across NAFLD, OB control and NW control. (B) Four-hour triglycerides during the AFTT across NAFLD, OB control and NW control. (C) Change in triglycerides from baseline to 4 h during the AFTT across NALFD, OB control and NW control. (D) Total area under the curve (tAUC) for baseline and 4 h triglycerides. Data are mean ± SD, designated by horizontal lines. Data points represent individual participant measurements. Groups with a shared letter indicate no statistically significant difference between groups. *p* < 0.05 is considered statistically significant. NAFLD, non-alcoholic fatty liver disease; NW, normal weight; OB, obese; tAUC, total area under the curve; TG, triglycerides.

Fasting and postprandial triglyceride concentrations are presented in Table 3 and Figure 1A-D. Results of a two-way ANOVA revealed a time ^ group interaction (*p* = 0.04), group effect (*p* = 0.006) and time effect (*p* < 0.0001) for triglycerides. In post hoc analyses, there were no group differences in fasting triglycerides (*p* > 0.05). The NAFLD and OB had 94% and 49% greater 4 h triglyceride concentrations than NW (*p* < 0.05), and although 4 h TG concentration was 25% higher in the NAFLD group than the OB group, this difference was not statistically significant (*p* > 0.05). Triglyceride tAUC was 75% greater for NAFLD compared with NW (*p* = 0.01) and 19% great than the OB group, though the latter comparison was not statistically significant (*p* > 0.05). The change in triglycerides from baseline to 4 h after the meal, Δtriglycerides, was 159% higher in the NAFLD group than NW (*p* = 0.03) and 67% higher than OB (*p* = 0.24). The Δtriglycerides for the OB was 55% more than for the NW (*p* = 0.08). There was a large effect size for the difference in postprandial triglycerides between the OB and NAFLD groups (*d* = 0.63), NW and OB groups (*d* = 0.84) and NW and NAFLD groups (*d* = 1.48). These effect size data suggest clinically significant differences among groups and suggest that a larger sample size may have revealed statistically significant differences among groups.

**FIGURE 2:**
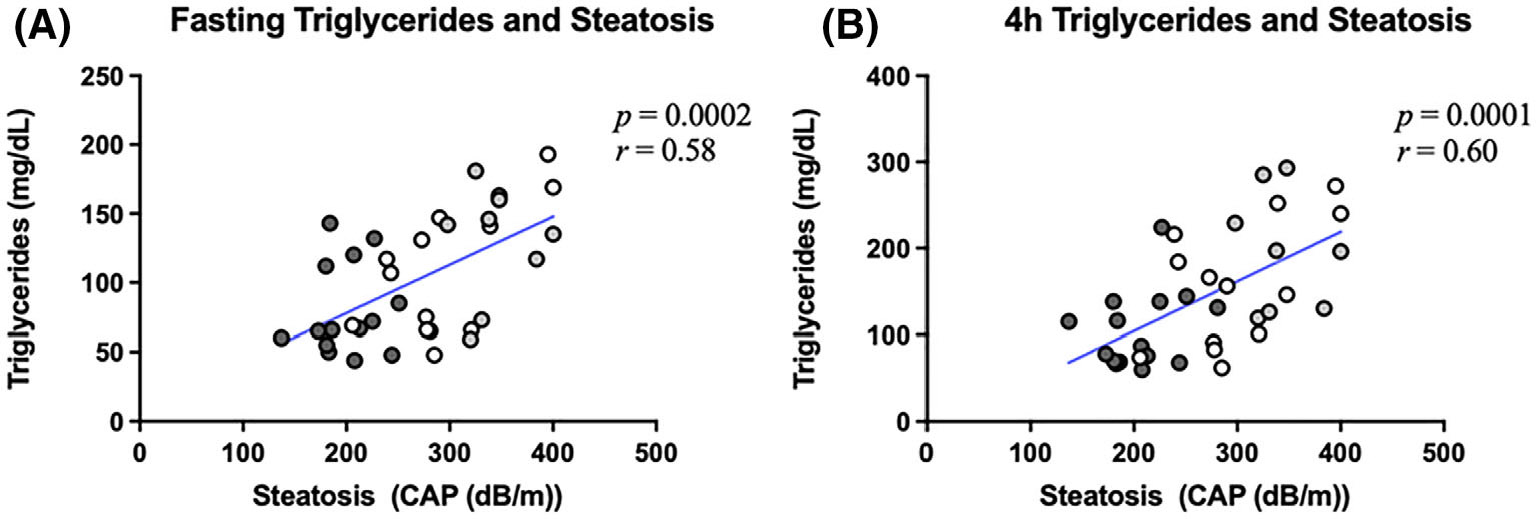
(A,B) Fasting and postprandial triglyceride correlations with steatosis. (A) Correlation between fasting triglycerides and steatosis in all groups. (B) Correlation between 4 h triglycerides and steatosis in all groups. Data points represent individual participant measurements. Results are based on results of Pearson correlation. Dark grey circles represent NW, white circles represent OB and light grey circles represent NAFLD. *p* < 0.05 is considered statistically significant.

Fasting triglycerides and 4 h postprandial triglycerides were positively correlated with steatosis (i.e., CAP score; *r* = 0.58, *p* = 0.0002; *r* = 0.60, *p* = 0.0001, respectively) (Figure 2A,B). Neither fasting (*r* = 0.06, *p* = 0.73) nor postprandial triglycerides (*r* = 0.24, *p* = 0.18) were correlated with fibrosis (i.e., kPa; data not shown).

**FIGURE 3:**
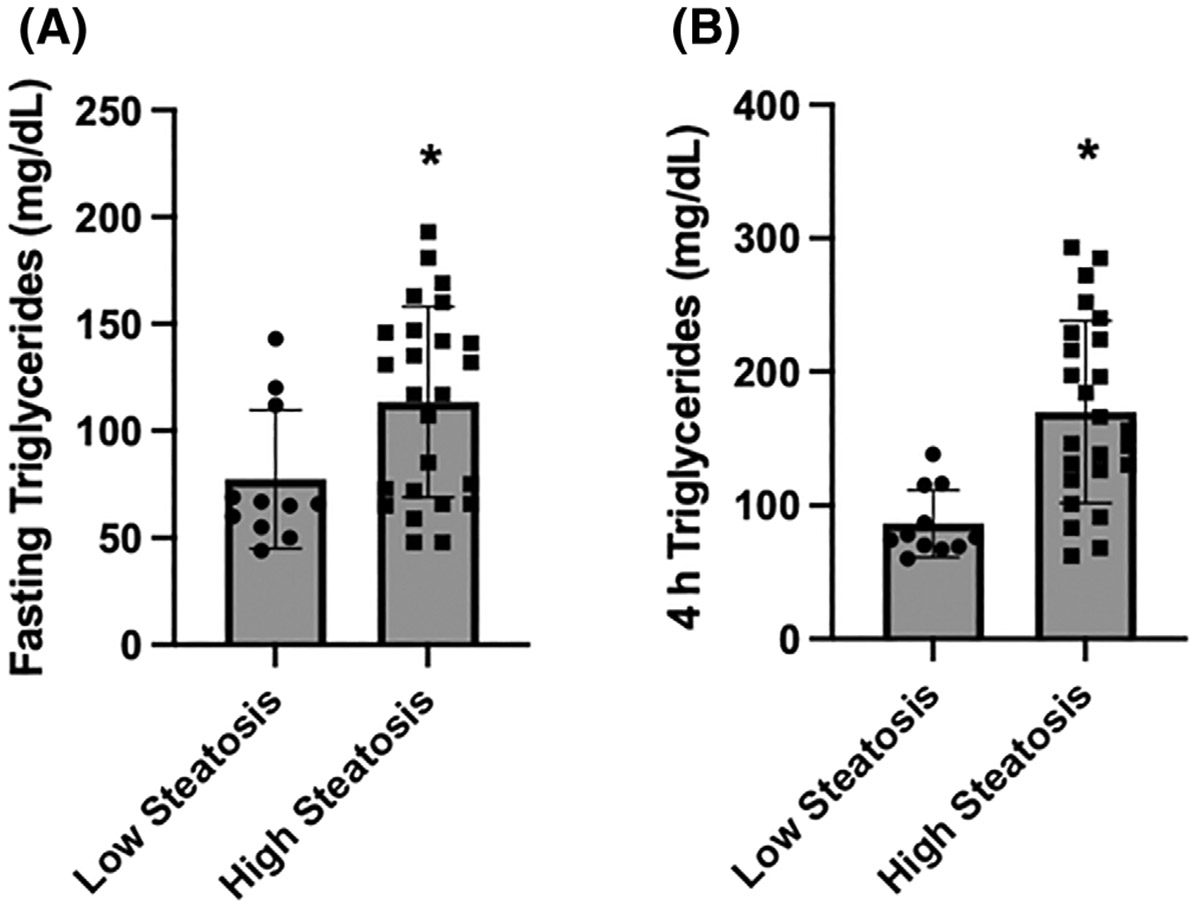
(A,B) Fasting and postprandial triglycerides and steatosis. (A) Fasting triglycerides in children with low steatosis and high steatosis. (B) The 4 h triglycerides in children with low steatosis and high steatosis. Data are mean ± SD. Asterisks indicates a statistically significant difference between groups. Data are based on results of an unpaired *t*-test. *p* < 0.05 is considered statistically significant.

To further examine the fasting and postprandial TG response in the context of steatosis, independent of group designation, we stratified all participants as either having a higher CAP (≥225 dB/m; *N* = 26; 5 NW, 12 OB, 9 NAFLD) or lower CAP (<225 dB/m; *N* = 11; 10 NW, 1 OB). With this approach, fasting triglycerides were 32% higher (*p* = 0.02) in participants with higher steatosis compared with participants with lower steatosis (Figure 3A). Similarly, participants with higher steatosis had 98% greater 4 h postprandial triglycerides compared with participants with lower steatosis (*p* < 0.0004; Figure 3B).

### 3.4 ∣ Fasting and postprandial FGF19

FGF19 concentrations and postprandial responses are displayed in Table 4. There were no differences among groups for fasting or postprandial FGF19 concentrations, the tAUC or ΔFGF19 (*p* > 0.05). There was a medium effect size for differences in postprandial FGF19 between the OB and NAFLD groups (*d* = 0.57) and the NW and OB groups (*d* = 0.47) and a large effect size between the NW and NAFLD groups (*d* = 0.80).

Fasting and postprandial FGF19 were not correlated with steatosis, fibrosis, or fasting and postprandial triglycerides (*p* > 0.05; data not shown). When participants were stratified by high and low steatosis, 4 h postprandial FGF19 was 150% higher in children with low steatosis compared with high steatosis (*p* < 0.05; Table 4). However, when participants were stratified by no fibrosis (kPa <6) and fibrosis (kPa >6), fasting FGF19 and 4 h postprandial FGF19 did not differ (*p* > 0.05).

### 3.5 ∣ Insulin, steatosis and postprandial triglycerides

NAFLD participants had higher fasting insulin than NW (mean difference = 43 μIU/mL; *p* < 0.0001) and OB (mean difference = 33 μIU/mL; *p* = 0.0005). Fasting insulin did not differ between OB and NW (mean difference = 10 μIU/mL; *p* = 0.30; data not shown).

**FIGURE 4:**
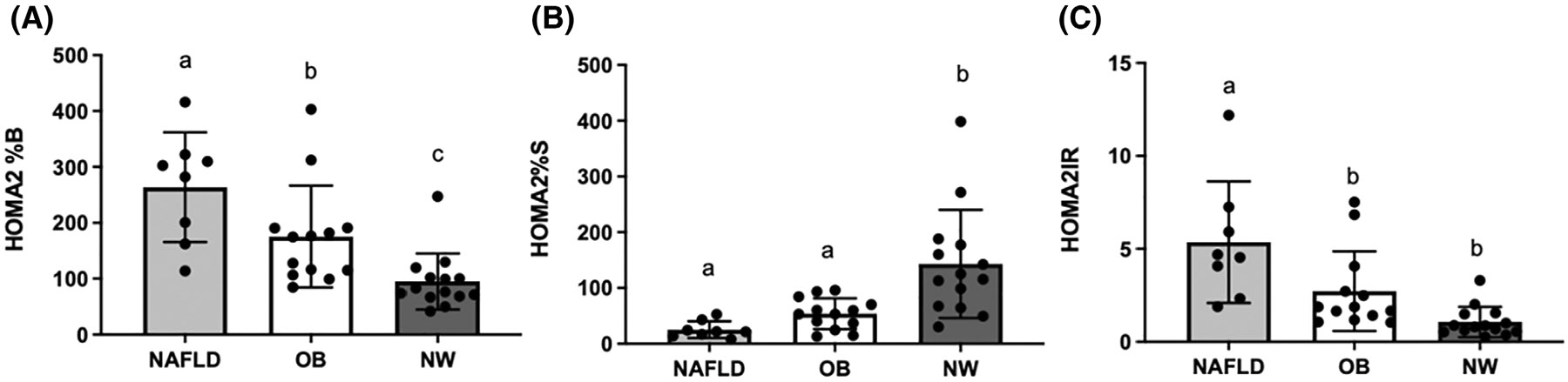
(A–C) HOMA2 measurements. (A) HOMA2%β at fasting across all groups. (B) HOMA2%S at fasting across all groups. (C) HOMA2IR at fasting across all groups. Data are mean ± SD; data points represent individual participant measurements. Data are based on results of a one-way ANOVA. Groups with a shared letter indicate no statistically significant difference between groups. NAFLD, non-alcoholic fatty liver disease; NW, normal weight; OB, obese. *p* < 0.05 is considered statistically significant.

NAFLD participants exhibited the highest modelled insulin secretion (HOMA2%B), followed by OB, then NW (*p* < 0.05) (Figure 4A). Insulin sensitivity, HOMA2%S, did not differ between NAFLD and OB, however NW had significantly higher insulin sensitivity than NAFLD and OB (Figure 4B; *p* < 0.05). Insulin resistance (HOMAIR) did not differ between OB and NW but was significantly lower in OB and NW compared with NAFLD (Figure 4C; *p* < 0.05).

Within the entire sample, when fasting insulin was stratified by higher and lower steatosis, participants with higher steatosis had ^4 greater fasting insulin (28 ± 21 μIU/mL) compared with participants with low steatosis (7 ± 4 μIU/mL; *p* = 0.003).

Fasting insulin was positively correlated with steatosis (i.e., CAP score; *r* = 0.80, *p* < 0.0001). Similarly, HOMA2IR was positively correlated with steatosis (*r* = 0.54, *p* = 0.0009). HOMA2IR was positively correlated with both fasting and 4 h triglycerides (*r* = 0.55, *p* = 0.0007; *r* = 0.54, *p* = 0.001, respectively; data not shown).

### 3.6 ∣ Diagnostic accuracy of metabolic and liver outcomes for NAFLD

**FIGURE 5:**
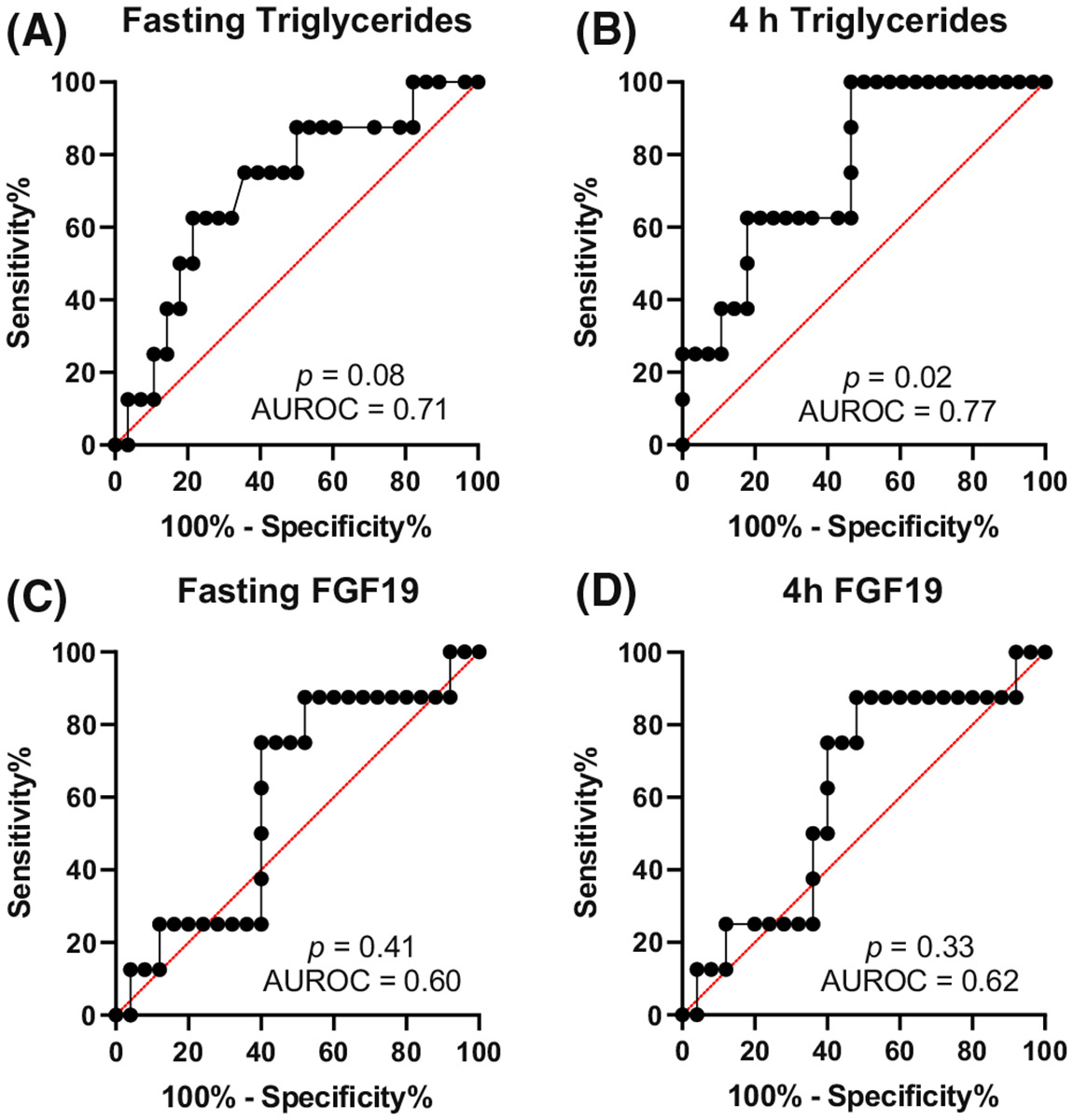
(A–D) AUROC plots. (A) AUROC for fasting triglycerides in all groups. (B) AUROC for 4 h triglycerides in all groups. (C) AUROC for fasting FGF19 in all groups. (D) AUROC for 4 h FGF19 in all groups. The red line denotes the line of no discrimination (AUROC of 0.5). A *p*-value >0.05 means that the AUROC is not significantly different from 0.5 and therefore there is not enough evidence to conclude that the test has diagnostic accuracy to distinguish between people with and without NAFLD. *p* < 0.05 is considered statistically significant.

Figure 5A-D show AUROC plots for fasting triglycerides, 4 h postprandial triglycerides, fasting FGF19 and 4 h postprandial FGF19. The pooled (all study participants) AUROC for fasting triglycerides, 4 h postprandial triglycerides, fasting FGF19 and 4 h postprandial FGF19 was 0.71 (95% confidence interval [CI]: 0.50–0.91; *p* = 0.08), 0.77 (95% CI: 0.60–0.94; *p* = 0.02), 0.60 (95% CI: 0.38–0.82; *p* = 0.41), 0.62 (95% CI: 0.40–0.83; *p* = 0.33), respectively.

**FIGURE 6:**
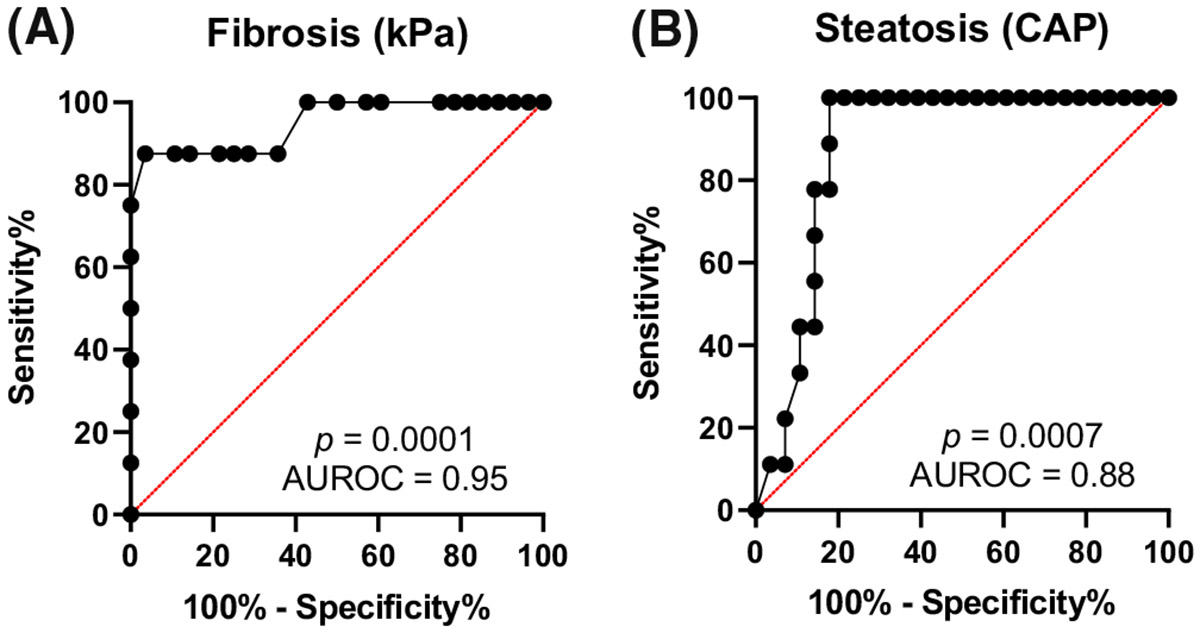
(A,B) AUROC plots. (A) AUROC for fibrosis (kPa) measured in all groups via Fibroscan. (B) AUROC for steatosis (CAP) measured in all groups via Fibroscan. The red line denotes the line of no discrimination (AUROC of 0.5). A *p*-value >0.05 means that the AUROC is not significantly different from 0.5 and therefore there is not enough evidence to conclude that the test has diagnostic accuracy to distinguish between people with and without NAFLD. *p* < 0.05 is considered statistically significant.

The pooled AUROC for fibrosis (kPa) and steatosis (CAP) was 0.95 (95% CI: 0.85–1.00; *p* = 0.0001) and 0.88 (95% CI: 0.77–0.99; *p* = 0.0007), respectively (Figure 6A,B).

## 4 ∣ DISCUSSION

In this study, we evaluated whether postprandial triglycerides and FGF19, within the context of an AFTT, could be used as screening tools for paediatric NAFLD. Our results show that postprandial triglycerides, but not postprandial FGF19, have moderate diagnostic accuracy for paediatric NAFLD. These data suggest that when using triglycerides as a screening option for paediatric NAFLD, measuring fasting concentrations may underestimate the presence of NAFLD and triglycerides measured after a high-fat meal can provide additional clinical insight for evaluating NAFLD risk. In this context, adolescents with obesity without diagnosed NAFLD demonstrated insulin resistance and steatosis, suggesting that adolescents with obesity warrant further screening for NAFLD. This notion is consistent with most^26,27^ but not all^4^ current paediatric NAFLD guidelines for standard of care.

Our findings support earlier work demonstrating that fasting triglycerides are not consistently elevated in adolescents with NAFLD or may be elevated in adolescents with obesity and insulin resistance, resulting in a lack of discrimination among patients with and without NAFLD.^7,28^ Meal tests (e.g., AFTT) provide metabolic challenges, that can reveal abnormal responses encountered in daily living that would otherwise be undetected if relying on fasting indices. For example, in the present study, while there were no group differences in fasting triglycerides, we observed a greater postprandial triglyceride response in adolescents with NAFLD compared with peers with normal weight, while children with obesity had an intermediate response. There are several factors that contribute to exaggerated postprandial triglyceride responses in adolescents, including obesity and insulin resistance. Notably, obesity and insulin resistance are key pathophysiological mechanisms involved in NAFLD and thus contribute to the complexity of identifying NAFLD independently of confounding metabolic comorbidities.

Obesity is commonly associated with NAFLD. Recent findings from Yu et al. demonstrate the estimated prevalence of NAFLD in children (mean age 13.2 years of age) with obesity was 29.4% in males and 22.6% in females.^28^ Therefore, determining the effect of obesity without NAFLD on postprandial triglycerides is important in determining the diagnostic accuracy of postprandial triglycerides as a screening tool for paediatric NAFLD. For example, similar to adults with NAFLD, adults with obesity alone have an exaggerated postprandial triglyceride response to a high-fat meal compared with adults without obesity.^22^ In the present study, adolescents with NAFLD had 67% higher triglyceride change from baseline to 4 h than adolescents with obesity and there was a large effect size for the difference in postprandial triglycerides between adolescents with NAFLD and adolescents with obesity. Although these findings were not statistically significant, they represent clinically relevant differences between children with NAFLD and children with obesity that suggest a larger sample size may have revealed statistically significant differences. The lack of statistical difference between the NAFLD and OB group was likely also related to our OB group having steatosis (e.g., >90% of adolescents with obesity had steatosis). Detecting steatosis in adolescents with obesity was not unusual as it has been reported that intra-hepatic triglyceride content increases proportionately with adiposity in adolescents and adults.^28,29^ When we stratified the study sample by higher and lower steatosis, we observed that adolescents with high steatosis, regardless of NAFLD diagnosis, had 97% higher postprandial triglycerides than adolescents with low steatosis. While fasting triglycerides were also higher in children with high steatosis, this was to a lesser degree (i.e., 32%) and may not be clinically meaningful because neither group had fasting triglycerides above the clinical threshold (i.e., 150 mg/dL). These data agree with previous findings showing that clinically normal fasting triglycerides frequently occur in both adolescents with NAFLD and adolescents with obesity.^7^ Thus, although not statistically significant, our finding that a large magnitude of difference in postprandial triglycerides and effect size was detected between adolescents with NAFLD and adolescents with obesity suggests that the study was underpowered, and that postprandial triglyceride assessment likely has promise for a new screening approach in paediatric NAFLD. This is important, as we also found that the adolescents with obesity in our study had steatosis but not fibrosis or diagnosed NAFLD, which highlights the underdiagnosis of paediatric NAFLD.

One prior study reported postprandial triglyceride responses in adolescents with and without NAFLD. Similar to our findings, Mager et al.^7^ observed that adolescents with NAFLD and adolescents with obesity both exhibited a greater postprandial triglyceride response than adolescents with normal weight, while postprandial triglycerides did not differ between adolescents with NAFLD and adolescents with obesity.^7^ In that study, the presence or absence of NAFLD was confirmed by biopsy in only a few patients and assessed by ultrasound in others. Abdominal ultrasound is only sensitive for NAFLD when hepatic triglyceride accumulation exceeds 30% of hepatocytes.^30^ Hepatic triglyceride accumulation of >5% determined via liver biopsy is the clinical threshold for a NAFLD diagnosis. Therefore, like our findings, adolescents with obesity without diagnosed NAFLD may have presented with steatosis suggestive of NAFLD despite a negative ultrasound. Moreover, Mager utilized a meal that was not scaled to body weight (i.e., 422 kcal for all study participants) and contained 43% fat (19% saturated fat), 39% CHO and 17% protein. These macronutrients ranges suggest that the test meal in Mager study contained 21 g of total fat. In comparison, the meal used in the present study was scaled to body weight (9 kcal/kg) and contained considerably more fat (69.5%), less CHO (20.9%) and less protein (9.5%), equating to ~75 g of total fat on average for the OB group (based on mean body mass [107.7 kg] in the OB group) in our study. The magnitude of the postprandial triglyceride response is directly related to the amount of total fat in the test meal, therefore the lack of difference observed by Mager could plausibly be related to insufficient quantities of fast in the test meal.^13^ Finally, the study by Mager contained a similarly small sample size (i.e., 11 NAFLD, 9 OB and 11 lean control) to our study. Therefore, although our findings somewhat agree with Mager, literature examining differences in postprandial triglycerides between adolescents with NAFLD, adolescents with obesity and adolescents with normal weight, may be limited by a small sample size and insufficient quantities of total fat in the test meal.

Epidemiological and cross-sectional data demonstrate that hepatic steatosis is consistently associated with insulin resistance, where insulin resistance can be both a cause and consequence of NAFLD.^31-33^ In fact, the presence of insulin resistance is a nearly ubiquitous feature of NAFLD and has been found to drive de novo lipogenesis and steatosis.^34^ In the present study, both adolescents with NAFLD and adolescents with obesity had lower insulin sensitivity and greater insulin secretion than adolescents with normal weight. Since several participants in the OB group had elevated insulin resistance and steatosis, the OB group represents a group with either high risk for NAFLD or potentially undiagnosed NAFLD in need of clinical assessment. We observed that insulin resistance was positively correlated with fasting and postprandial triglycerides and hepatic steatosis, supporting that insulin resistance is a core component linking postprandial triglycerides to hepatic triglyceride accrual in paediatric NAFLD.

Prior studies of FGF19 in paediatric NAFLD have focused on fasting concentrations and not postprandial responses to a high-fat meal.^35^ FGF19 production and secretion is increased following meal intake and bile acid entry into the small intestine, and thus FGF19 acts principally in the post-absorptive state.^36^ We expected that postprandial FGF19 would, like triglycerides, show different responses among groups. While we did not observe a difference in fasting or postprandial FGF19 among groups, postprandial FGF19, but not fasting FGF19, was 150% higher in adolescents with low steatosis compared with high steatosis. However, unexpectedly, neither fasting nor postprandial FGF19 were associated with any triglyceride parameter, steatosis or fibrosis. Our results on fasting FGF19 agree with previous studies reporting that children with high steatosis exhibited lower fasting FGF19 compared with children with low steatosis.^17^ While findings from others have demonstrated an inverse correlation between fasting FGF19 and hepatic damage in adolescents with NAFLD, to our knowledge, postprandial FGF19 has not been studied in adolescents with obesity with and without NAFLD.^35^ Research in adults with NAFLD may provide some insight. Adults with NAFLD exhibit altered postprandial FGF19 signalling following a standardized high-fat meal (i.e., 30 g cream per square meter body surface area), sometimes referred to as “FGF19 resistance,” marked by an inability of FGF19 to suppress 7α-hydroxy-4-cholesten-3-one (C4), a metabolic intermediate involved in the rate-limiting step for bile acid synthesis.^37^ Therefore, adults with NAFLD may experience reduced postprandial FGF19 production and signalling, leading to reduced triglyceride disposal in the liver, and increased postprandial triglycerides. A major role of FGF19 is to promote FFA oxidation (e.g., via inhibiting acetyl-CoA carboxylase) and inhibit triglyceride synthesis in the liver via suppressing SREBP1c.^38,39^ Our finding that adolescents with high steatosis had lower postprandial FGF19 than children with low steatosis support further explorations of the link between postprandial FGF19 and hepatic fat accrual, such that impairments in FGF19 production or signalling at the site of the liver may lead to on-going triglyceride synthesis.

Historically, liver enzymes have been the gold standard for screening in adolescents suspected of NAFLD. Once abnormal liver enzymes are detected and other co-morbidities or liver diseases have been ruled out, adolescents with persistently elevated ALT often undergo an imaging-related screening tool, including either ultrasound, elastography or MRI.^40^ These screening methods are problematic for the early detection of NAFLD, though, because liver enzymes can fall within normal ranges in up to 70% of children with NAFLD and can increase for reasons beyond NAFLD, including medication, stress and other liver diseases.^41^ In the present study, fasting triglycerides, fasting FGF19 and postprandial FGF19 all had low diagnostic accuracy (e.g., could not discriminate between adolescents with and without NAFLD). Of the biochemical tests performed in the present study, postprandial triglycerides concentration had the strongest diagnostic accuracy, though this measure was moderately accurate. Fibroscan measurements, including steatosis (CAP) and fibrosis (kPa), both had strong diagnostic accuracy; however, Fibroscan is expensive and not widely accessible. Finally, NAFLD patients were referred for the present study based on high liver enzymes (e.g., ALT) so comparing the diagnostic accuracy of ALT to postprandial triglycerides and FGF19 was not appropriate. The clinical challenge is to distinguish adolescents with obesity, in whom insulin resistance and steatosis are common as shown in our cohort, from those with similar characteristics and more advanced liver disease that meets the definition of NAFLD. We acknowledge that this preliminary study had a small sample size and the true diagnostic accuracy of postprandial triglycerides for NAFLD may be stronger or weaker when tested in a larger cohort. To our knowledge, this study is among the first to report the diagnostic accuracy of both fasting and postprandial triglycerides and FGF19 in paediatric NAFLD. Therefore, our finding that postprandial triglycerides have moderate diagnostic accuracy for paediatric NAFLD is an important step toward creating a novel, accurate set of biomarkers for this disease.

One limitation of our study was that most of the adolescents with obesity without diagnosed NAFLD were above the reported threshold for hepatic steatosis and had evidence of insulin resistance, both of which overlapped with the NAFLD group, which may have made it more difficult to distinguish those groups on some measurements. Insulin resistance is common in adolescents with obesity, but the prevalence of hepatic steatosis is likely underestimated in the general population. However, the NAFLD group was clearly different from the OB group in their liver fibrosis assessment and liver enzymes. Nevertheless, the AFTT as a screening tool for paediatric NAFLD may be confounded by metabolic comorbidities, including obesity and insulin resistance. This is an important consideration to make in future work because both obesity and insulin resistance can increase postprandial triglycerides independently of a NAFLD diagnosis. Further, FGF19 usually peaks around 2–3 h post-meal intake. We collected blood samples at baseline and 4 h post-meal, therefore we may not have captured the postprandial FGF19 peak. Collecting more samples over a longer time may also help confirm the optimal time for measuring postprandial triglycerides or other biomarkers for paediatric NAFLD.

In summary, these preliminary data demonstrate that postprandial triglyceride concentrations following a high-fat meal could be used as a screening tool for paediatric NAFLD. Our data show that steatosis may be common in adolescents with obesity, regardless of NAFLD diagnosis, and thus screening for NALFD in adolescents with obesity should be considered. We also found that the postprandial rise in FGF19 was blunted in adolescents with higher liver steatosis; however, FGF19 measures did not have diagnostic accuracy for NAFLD. Future work should determine when peak concentrations of both FGF19 and postprandial triglycerides occur in adolescents with NAFLD to more accurately determine the diagnostic accuracy of postprandial triglycerides and FGF19 for paediatric NAFLD.

## Figures and Tables

**TABLE 1:** Participant characteristics

	NAFLD	OB	NW	*p*-value
Participants (*n*)	9	13	15	—
Sex (M/F)	7/2	5/8	6/9	—
Age (years)	15 ± 2	17 ± 2	17 ± 2	0.11
Height (in)	66 ± 3	68 ± 3	66 ± 5	0.59
Weight (lb)	238 ± 62^a^	237 ± 39^a^	135 ± 23^b^	**<0.0001**
BMI (kg/m^2^)	37 ± 8^a^	37 ± 6^a^	21 ± 3^b^	**<0.0001**
Systolic BP (mmHg)	124 ± 11^a^	128 ± 7^ab^	117 ± 9^a^	**0.01**
Diastolic BP (mmHg)	67 ± 8	73 ± 8	67 ± 7	0.09
Body fat (%)	48 ± 6^a^	48 ± 6^a^	31 ± 10^b^	**<0.0001**
Android body fat (%)	56 ± 7^a^	55 ± 7^a^	28 ± 12^b^	**<0.0001**
Gynoid body fat (%)	46 ± 5^a^	49 ± 6^a^	34 ± 11^ab^	**0.0001**
Trunk fat (kg)	28.9 ± 13.3^a^	26.9 ± 6.6^a^	7.9 ± 3.3^b^	**<0.0001**
Fibrosis score (kPa)	10.1 ± 5.5^a^	5.3 ± 0.9^b^	5.0 ± 0.7^b^	**0.0002**
Steatosis score (CAP, dB/m)	343 ± 34^a^	300 ± 59^a^	205 ± 36^b^	**<0.0001**

*Note*: Data are mean ± SD. The *p*-value column reflects the results of a one-way ANOVA. Within rows, groups with shared letters indicate no statistically significant difference between groups in post hoc analyses. *p* < 0.05 is considered statistically significant and indicated in bold.

Abbreviations: BMI, body mass index; BP, blood pressure; CAP, controlled attenuation parameter; NAFLD, non-alcoholic fatty liver disease; NW, normal weight; OB, obese.

**TABLE 2:** Fasting metabolic and liver biochemistries

	NAFLD	OB	NW	*p*-value
Cholesterol (mg/dL)	153 ± 19	156 ± 28	164 ± 30	0.59
HDL-C (mg/dL)	41 ± 7^a^	42 ± 14^a^	60 ± 13^b^	**0.0004**
LDL-C (mg/dL)	87 ± 16	91 ± 19	88 ± 21	0.92
VLDL-C (mg/dL)	25 ± 8^a^	23 ± 9^ab^	16 ± 6^b^	**0.02**
HbA1c (%)	5.4 ± 0.3^ab^	5.4 ± 0.4^a^	5.0 ± 0.4^b^	**0.02**
Insulin (μIU/mL)	51 ± 32^a^	18 ± 8^b^	8 ± 4^b^	**<0.0001**
Adiponectin (ng/mL)	2268 ± 356	2388 ± 727	2853 ± 900	0.19
ALT (U/L)	108 ± 92^a^	26 ± 11^b^	20 ± 12^b^	**0.0001**
AST (U/L)	71 ± 51^a^	28 ± 8^b^	31 ± 12^b^	**0.0009**

*Note*: Data are mean ± SD. The *p*-value column reflects the results of a one-way ANOVA. Within rows, groups with shared letters indicate no statistically significant difference between groups. *p* < 0.05 is considered statistically significant and indicated in bold.

Abbreviations: ALT, alanine aminotransferase; AST, aspartate aminotransferase; HbA1c, haemoglobin A1c; HDL-C, high-density lipoprotein cholesterol; LDL-C, low-density lipoprotein cholesterol; NAFLD, non-alcoholic fatty liver disease; NW, normal weight; OB, obese; VLDL-C, very low-density lipoprotein cholesterol.

**TABLE 3:** Triglyceride parameters

	NAFLD	OB	NW	*p*-value
Fasting TG (mg/dL)	127 ± 42	115 ± 47	79 ± 32	>0.05
Postprandial TG (mg/dL)	197 ± 69^a^	157 ± 72^a^	105 ± 45^b^	<0.05
tAUC TG (mg/dL)	647 ± 217^a^	544 ± 230^ab^	369 ± 139^b^	0.006
ΔTG (mg/dL)	70 ± 37^a^	42 ± 40^ab^	27 ± 35^b^	0.04

*Note*: Data are mean ± SD. The *p*-value column reflects the results of either a one-way or two-way ANOVA. Within rows, groups with shared letters indicate no statistically significant difference between groups in post hoc analyses. *p* < 0.05 is considered statistically significant.

Abbreviations: NAFLD, non-alcoholic fatty liver disease; NW, normal weight; OB, obese; tAUC, total area under the curve; TG, triglycerides.

**TABLE 4:** FGF19 before and after the high-fat meal

	NAFLD	OB	NW	*p*-value
Fasting FGF19 (pg/mL)	539 ± 382	591 ± 623	1866 ± 2061	>0.05
Postprandial FGF19 (pg/mL)	465 ± 330	679 ± 648	1809 ± 2086	>0.05
tAUC FGF19 (pg/mL)	2007 ± 1418	3927 ± 5402	7293 ± 8318	0.21
ΔFGF19 (pg/mL)	−17 ± 175	257 ± 555	−112 ± 409	0.14
	CAP ≤ 220	CAP ≥ 220	—	*p*-value
Fasting FGF19 (pg/mL)	1301 ± 1505	577 ± 499	—	0.08
4 h postprandial FGF19 (pg/mL)	1537 ± 1299^a^	616 ± 672^b^	—	0.03
	kPa <6	kPa >6	—	*p*-value
Fasting FGF19 (pg/mL)	1713 ± 1997	597 ± 561	—	0.11
4 h postprandial FGF19 (pg/mL)	1809 ± 2111	393 ± 279	—	0.06

*Note*: Data are mean ± SD. The *p*-value column reflects the results of a one-way, two-way ANOVA or unpaired *t*-test. Within rows, groups with shared letters indicate no statistically significant difference between groups. *p* < 0.05 is considered statistically significant.

Abbreviations: NAFLD, non-alcoholic fatty liver disease; NW, normal weight; OB, obese; tAUC, total area under the curve; TG, triglycerides.
